# Wear, fatigue characteristics, and antibacterial performance of UHMWPE reinforced with HAP+TiO_2_ for biomedical applications

**DOI:** 10.1038/s41598-026-58449-3

**Published:** 2026-06-21

**Authors:** Ahmed Salama, T. A. Osman, R. M. Rashad, Bahaa M. Kamel, M. M. Salem

**Affiliations:** 1 Department of Manufacturing Engineering and Production Technology, Modern Academy for Engineering and Technology in Maadi, Cairo, 12622 Egypt; 2 Mechanical Design and Production Engineering Department, Cairo University, Giza, 12613 Egypt; 3 Mechanical Engineering Department, National Research Centre, Giza, Egypt

**Keywords:** Ultra-high molecular weight polyethylene, Hydroxyapatite, Biomedical, Artificial joints, Bio tribology, Wear-resistance, Engineering, Health care, Materials science, Medical research, Nanoscience and technology

## Abstract

This study aimed to develop and evaluate hybrid composites of ultra-high molecular weight polyethylene (UHMWPE) reinforced with hydroxyapatite (HAP) and titanium dioxide (TiO_2_) for synthetic cartilage applications in joint prostheses. Three composite formulations were prepared using a solvent dispersing method followed by hot pressing, and their mechanical, tribological, and antibacterial properties were analyzed. Transmission electron microscopy (TEM) and X-ray diffraction (XRD) were employed to assess the dispersion quality of the fillers. TiO_2_ content was varied (1, 3, 5, and 10 wt%) while HAP content was fixed at 20 wt%. In the UHMWPE matrix, HAP served as a bone osteo-inductive agent and TiO_2_ as an anti-wear additive. Wear testing against Ti6Al4V alloy using dry sliding and simulated body fluid (SBF) revealed that 3 wt% TiO_2_ enhanced wear resistance due to the formation of a dense lubricant film. The Agar Well Diffusion method confirmed significant antibacterial activity in the composites. Furthermore, fatigue tests conducted at frequencies of 1 Hz and 3 Hz demonstrated improved fatigue life and maximum stress for the P-H-3T composite (3 wt% TiO_2_). This improvement was attributed to the homogeneous dispersion of the nanoparticles, which facilitated better stress transfer and delayed crack initiation. Excessive TiO_2_ led to particle agglomeration, reducing fatigue resistance. The results highlight that 3 wt% TiO_2_ provides an optimal balance between strength, wear resistance, and fatigue life for biomedical applications.

## Introduction

Recently, the use of Ultra-high molecular weight polyethylene (UHMWPE) in artificial joint replacement has become increasingly popular because of its exceptional mechanical and wear resistance properties, which exceed those of other polymers^1,2^. UHMWPE is a self-lubricating material with high chemical stability and biocompatibility^3,4^. Its performance has been greatly improved in order to give young and active patients long-lasting implants. A polymer liner was used to replace the degraded cartilage in the late 1950s. Sir John Charnley used polytetrafluoroethylene (PTFE), a polymer with low friction properties that was able to articulate with a metallic femoral head, to replace the natural acetabulum in hip replacement surgery. However, the initial “low friction arthroplasties” project was unsuccessful after a few years of implantation due to the unusually low wear resistance of PTFE. In 1962, UHMWPE (a comparable low-friction polymer that is substantially more wear-resistant than PTFE) was used as a substitute for PTFE, yielding noticeably superior outcomes. Since then, major advancements in arthroplasty have been made, but UHMWPE continues to be an excellent standard for artificial knees, shoulders, and hips^5,6^.

It is important to distinguish between metallic-based and polymer-based composites in biomedical applications to clarify the rationale behind the present material selection. Metallic biomaterials such as titanium alloys and cobalt–chromium systems are widely used for load-bearing implants due to their high strength and stiffness. Titanium alloys typically exhibit an elastic modulus of approximately 100–110 GPa, while cobalt–chromium alloys may reach 210–230 GPa, compared to natural cortical bone, which ranges between 7 and 30 GPa. However, this significant stiffness mismatch may lead to stress shielding effects and long-term bone resorption. In contrast, polymer-based composites such as UHMWPE exhibit much lower elastic modulus values (approximately 0.5–1 GPa), lower density, improved impact resistance, superior wear behavior in articulating components, and better shock absorption characteristics. In joint replacement systems, metallic components are typically employed as structural supports, while polymeric materials serve as bearing surfaces where tribological performance becomes critical. The incorporation of bioactive fillers such as HAP enhances osteoconductivity, while TiO₂ contributes to improved wear resistance and antibacterial functionality. Therefore, the present hybrid UHMWPE-based composite is not intended to replace metallic load-bearing implants but rather to optimize articulating biomedical components where fatigue–wear resistance and biological response are simultaneously required. This distinction underscores the clinical relevance of the developed system in orthopedic and joint prosthesis applications^5^.

Although improving the tribological properties of UHMWPE is a high priority, debris formed by the abrasion of the polyethylene polymer harmed the material’s mechanical stability^7,8^. UHMWPE polymer composites usually employ nano-fillers to enhance their functional characteristics^9–11^. Recent studies have extensively explored the technique of incorporating nano-fillers into a polyethylene polymer matrix for reinforcement. Recently, research has shifted from single-filler reinforcement toward hybrid systems to achieve a multi-functional response. The synergy between bio-active ceramics and metallic oxides has been identified as a robust strategy to overcome the inherent trade-offs between mechanical strength and long-term durability. For instance^12^, demonstrated that incorporating hybrid nano-fillers facilitates the formation of a stable tribological transfer film, which significantly reduces surface degradation. This synergistic effect is crucial for biomedical applications, where a balance between high load distribution, enhanced fatigue resistance, and antibacterial efficacy (fatigue–wear–antibacterial synergy) is required to ensure implant longevity. Recent studies in 2024 and 2025^13,14^ further emphasize that this integration significantly boosts antibacterial efficacy and mechanical stability. Following these synergistic trends, previous studies showed that by adding 1.0 wt% GO nanosheets^8^, the wear resistance greatly increased. Fahad Alam et al.^15^ reinforced UHMWPE in 2015 by adding ZnO and Ag. Selim Gürgen et al.^4^ in 2019 examined PTFE and aramid fibers, while in 2020, Gürgen et al.^9^ employed SiC. In 2021, Xueqin Kang et al.^3^ investigated epigallocatechin gallate’s effect on biological behavior. Fatigue behavior plays a critical role in the performance of biomedical materials, especially those used in artificial joints, where cyclic loading is prevalent. This study investigates the fatigue resistance of UHMWPE composites reinforced with HAP and TiO2, two additives known for their bone osteo-inductive and anti-wear properties. Fatigue tests, conducted at different frequencies, complement wear and antibacterial assessments, providing a comprehensive evaluation of the composite’s suitability for long-term biomedical applications.

When nanoparticles are dispersed as fillers in nanocomposites, they exhibit notably superior properties compared to pure polymers or conventional composites, as has been highlighted in several studies^16–18^. The addition of filler materials, such as nanoparticles (NPs), to the polymer matrix can enhance the tribological performance of UHMWPE. For biological applications, it is crucial to choose a filler substance that may improve mechanical and tribological properties while also inhibiting bacterial growth. Titanium dioxide (TiO_2_) meets these requirements because of its inert property and has an inhibitory action that prevents microorganism proliferation^19–22^. Due to its beneficial qualities, which include excellent corrosion resistance, low toxicity, high strength, and superior fatigue resistance, (TiO_2_) is commonly employed in biomaterials. In order to enhance the performance of biomaterials, surface modification techniques such as anodization have been utilized to improve cell adhesion, proliferation, and differentiation, as well as antibacterial capacity, as noted in previous studies^23^. In 2011, Denis Mihaela et al.^24^ examined the impact of two TiO_2_ phases (rutile and anatase) on the polymer characteristics of UHMWPE. The composite’s mechanical characteristics significantly enhanced as a result of the better TiO_2_ nanoparticle dispersion in the polymer matrix. Aside from the anatase TiO_2_ phase which exhibits a significantly higher elastic modulus, polyethylene composites containing both anatase and rutile TiO_2_ did not exhibit any discernible variations in their mechanical or morphological characteristics. In 2017, G. Celebi et al.^20^ investigated into the characterization of UHMWPE-TiO_2_ composites created using the gelation/crystallization method. TiO_2_ was added in various weight percentages (0.5, 1, and 2wt%) in Celebi’s study. The results showed that TiO_2_ particles adhered well to the UHMWPE matrix. Furthermore, TiO_2_ particles acted as nucleation agents for UMMWPE chains, increasing crystallinity as well as the elastic modulus of UHMWPE.

In orthopedic applications, ceramics have been employed as implants or coatings for implants. due to their strong chemical biocompatibility and structural resemblance to bone minerals. The most popular materials for replacing bone are hydroxyapatite and tricalcium phosphate (TCP)^25–29^. By offering a porous structure comparable to that of real bone along its surface or within its pores, hydroxyapatite encourages the growth of bone cells^30,31^. When combined with a polymer, calcium phosphate ceramics (HAP) have a more adaptable biodegradability than other ceramics, which increases the material’s bioactivity. Furthermore, as a hydroxyapatite scaffold dissolves in the body, calcium and phosphate ions are released, stimulating the formation of new bone. One of the most significant benefits of hydroxyapatite for bone scaffold applications is its low immunogenicity and toxicity^32^. S. V. Panin et al.^33^ investigated the effect of varying the size of HAP particles in the polymer matrix of UHMWPE on dry sliding wear resistance in 2012. The addition of HAP nanoparticles in the range of 0.1-0.5wt.% caused a significant change. Adding 20% weight of micron size HAP, on the other hand, has the same effect. Seyed A Mirsalehi et al.^34^ reported the biocompatibility properties of UHMWPE reinforced with nano-hydroxyapatite in 2015. HAP was added in concentrations ranging from 0% to 50% by weight, with the results indicating that 20% by weight is more appropriate for spongy bone replacement.

**Fig. 1:**
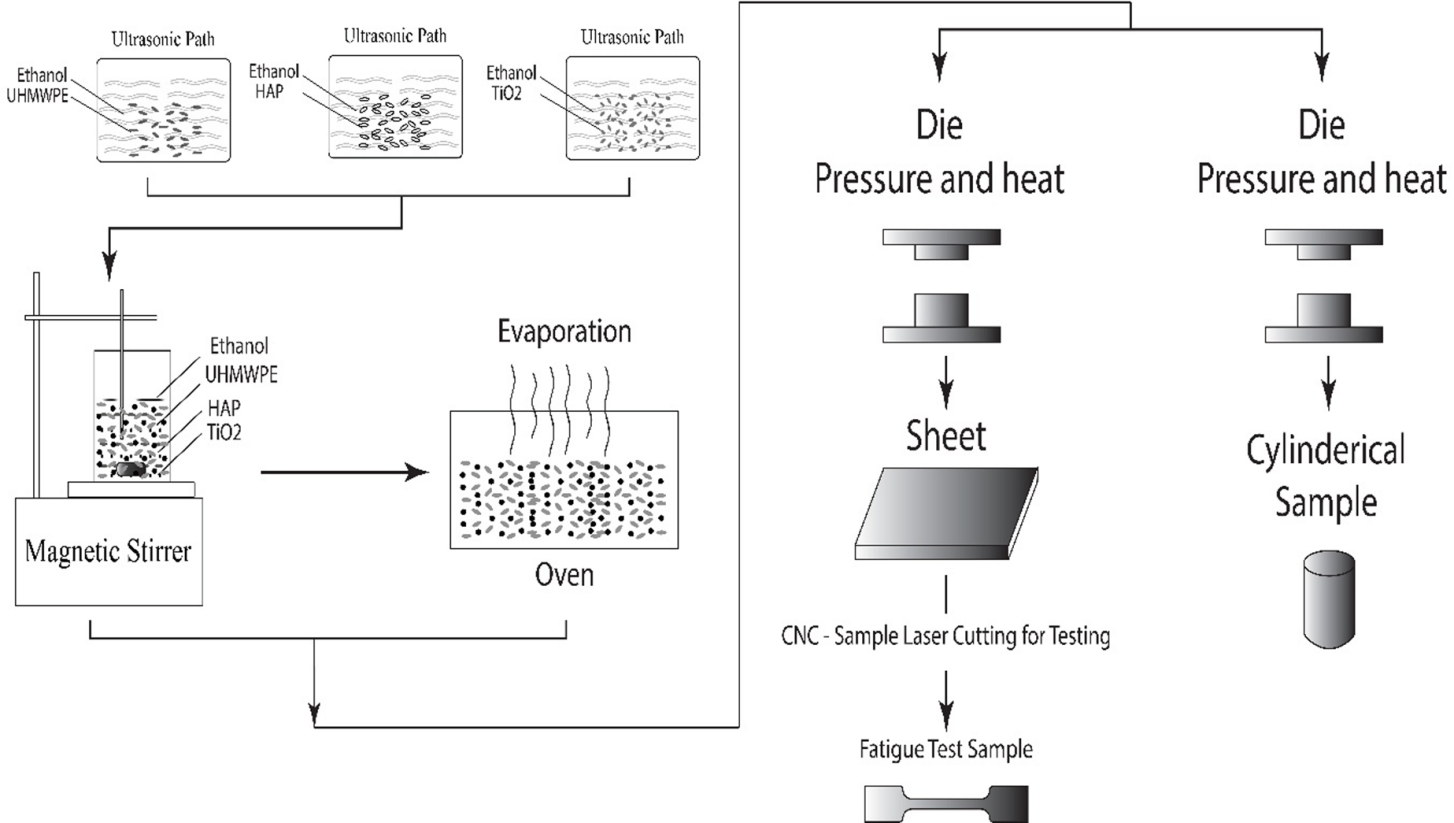
Schematic view for the sample fabrication.

Despite researchers having studied extensively the enhancement of the tribological properties of UHMWPE-based composites in biomedical polymers by adding various types of fillers, there has been little investigation into the joint impact of anti-wear and bone osteo-induction additives on the tribological performance of UHMWPE in this field. The UHMWPE matrix in this work has been loaded with nano-TiO_2_ (anti-wear) and HAP (bone osteo-induction) fillers. Denis Mihaela et al.^24^ reported that the TiO_2_ NPs saturation level was greater than 0.5 wt% for polymers with single carbon chains such as UHMWPE, HDPE, and LDPE. In this regard, the polymer matrix was supplemented with TiO_2_ at different percentages (1, 3, 5, and 10%), and the wear performance of each was examined in order to establish the optimum amount that yields the lowest composite wear rate. Furthermore, as reported in^34–36^, a fixed percentage of HAP 20wt.% was added to the UHMWPE matrix as an optimal percentage. The solvent dispersing methodology, as shown in Fig. 1, was used in this work, and will be discussed further below. The outcome mixture is more homogeneous, and the additives are well distributed into the polymer matrix when utilizing the solvent dispersing methodology compared to the conventional twin screw mixer result, as seen in the SEM photos^37^. The material characteristics were checked by Transmission electron microscopy (TEM), X-ray diffraction (XRD) as shown in the Sect. 3. Tribological tests were discussed in Sect. 4, and antibacterial testing was performed and discussed in Sect. 5.

Despite the documented benefits of these individual fillers, there remains a significant gap in understanding how advanced manufacturing pathways—specifically Direct Powder Extrusion (DPE)—interact with these hybrid systems. This work fills this gap by investigating the HAP/TiO₂ synergy within DPE-printed components, aligning the structural optimization of additive manufacturing with the latest trends in high-performance bioactive composites. This study investigates the mechanical integrity and fatigue resistance of these fabricated composites, providing a comprehensive evaluation of the system’s suitability for long-term biomedical applications.

## Experimenting

### Materials

**Fig. 2:**
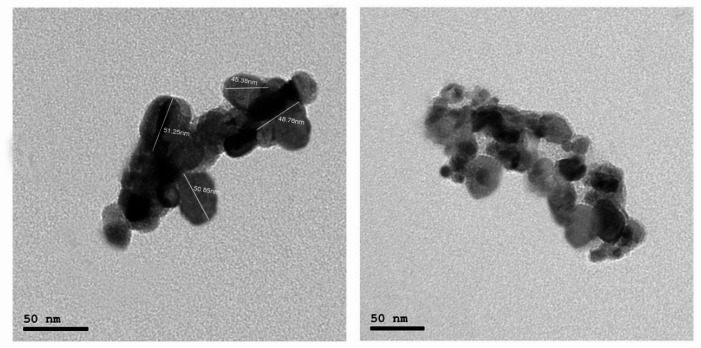
TEM image for TiO_2_.

Polyethylene with the chemical formula (C₂H₄)ₙ, ultra-high molecular weight (Mw = 3,000,000–6,000,000), and a density of 0.94 g/mL at 25 °C was used as the polymer matrix material. Hydroxyapatite (HAP, Ca₅(OH)(PO₄)₃) with a molecular weight of 502.31 g/mol and an average particle size of 2.5 μm was used as a bioactive filler. Titanium dioxide nanoparticles (TiO₂) with an average particle size of 50 ± 5 nm and a density of approximately 4.23 g/cm³ were also incorporated as reinforcement. The morphology and particle size of TiO₂ nanoparticles were confirmed using transmission electron microscopy (TEM, JEM-2100 JEOL, Japan), as shown in Fig. 2. Ethanol (99.9% purity) was used during the preparation process. All materials were purchased from Sigma Aldrich.

For the wear tests, a simulated body fluid (SBF) based on Hank’s solution was employed as the lubricant, and its composition is presented in Table 1^49^. The solution was selected because its ionic composition and physiological pH closely resemble human body fluid conditions. The pH of the prepared solution was maintained at approximately 7.4 ± 0.1 during the tribological experiments. The counterface disc used in the wear tests was fabricated from Ti6Al4V alloy.

**Table 1:** Simulated body fluid composition (Hank’s solution).

Prepare 800 mL of distilled water in a suitable container, then add:		
**Name**	**Formula**	**g/l**
Sodium chloride (mw: 58.44 g/mol)	NaCl	8
Potassium Chloride (mw: 74.55 g/mol)	KCl	0.4
Calcium Chloride (mw: 110.98 g/mol)	CaCl_2_	014
Magnesium Sulfate Heptahydrate (mw: 246.47 g/mol)	MgSO_4_−7H_2_O	0.1
Magnesium Chloride Hexahydrate (mw: 203.30 g/mol)	MgCl_2_−6H_2_O	0.1
Sodium Phosphate Dibasic Dihydrate (mw: 177.99 g/mol)	Na_2_HPO_4_−2H_2_O	0.06
Potassium Phosphate Monobasic (mw: 136.09 g/mol)	KH_2_PO_4_	0.06
D-Glucose (Dextrose) (mw: 180.16 g/mol)	C_6_H_6_O_6_	1
Sodium Bicarbonate (mw: 84.01 g/mol)	NaHCO_3_	0.35
Add distilled water until the volume is 1 L		

### Sample fabrication

Six samples were prepared for testing, five of which contained a fixed HAP content (20 wt%) combined with four different TiO₂ concentrations (1, 3, 5, and 10 wt%). The sixth sample consisted of neat UHMWPE used as a reference material. The samples were fabricated in two stages. In the first stage, the composite powders were blended using a solvent dispersing technique according to the compositions listed in Table 2, following the methodology described in our previous work^37^. Initially, different concentrations of TiO₂ and HAP were ultrasonically dispersed in 50 mL of ethanol for 1 h, while UHMWPE powder was separately dispersed in 100 mL of ethanol for the same duration. Subsequently, the mixed suspension underwent an additional hour of ultrasonic treatment to improve nanoparticle distribution within the UHMWPE matrix. To minimize particle precipitation and improve homogeneity, the mixture was magnetically stirred for 45 min at 70 °C. Before the molding stage, the suspension was heated at 90 °C for 5 h to ensure complete solvent evaporation.

The dried composite powders were then compression molded using a stainless-steel mold with dimensions of 100 mm × 100 mm × 5 mm to produce the composite sheets required for subsequent mechanical, tribological, and fatigue testing. The evaporation temperature of 90 °C was chosen to thoroughly eliminate residual solvent while remaining safely below the melting temperature $\:({T}_{m}\approx\:135-145\:{}^{o}C)$ and thermal degradation threshold (> 400 $\:{}^{o}C)$ of the UHMWPE matrix, thereby preserving its inherent chemical properties.

**Fig. 3:**
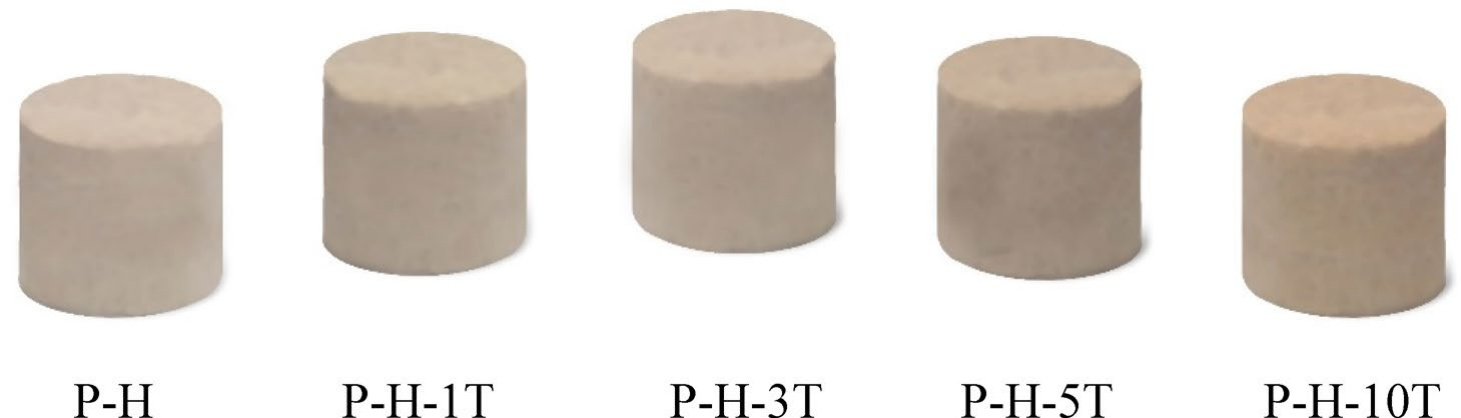
Cylindrical samples for the wear test.

The second stage involved the use of a hot compression molding process to create Nano composite cylindrical samples for wear testing. The degradation temperature of the polymer was observed to be above 400 °C based on a thermogravimetric (TGA) analysis for a neat polymer (UHMWPE) specimen^37^. As a result, the composite compaction temperature should be less than 400 °C. In this regard, the previously described mixture was put into a 150 °C pre-heated mold and compressed in an electrically heated press to a pressure up to 150 kg/cm^2^. After that, the temperature was raised to 250 °C (over the melting point) and kept there for 10 min. Then cooled at a rate of 15 °C/min after the hot pressing. As shown in Fig. 3, the samples were obtained as cylindrical objects with a 15 mm diameter and 15 mm length.

**Table 2:** Different samples composition.

No.	Sample ID	Composition	Weight for 150 g Samples		
			UHMWPE	HAP	TiO_2_
1	P	100% UHMWPE	150	0	0
2	P-H	80% UHMWPE + 20% HAP	120	30	0
3	P-H-1T	79% UHMWPE + 20% HAP + 1% TiO_2_	118.5	30	1.5
4	P-H-3T	77% UHMWPE + 20% HAP + 3% TiO_2_	115.5	30	4.5
5	P-H-5T	75% UHMWPE + 20% HAP + 5% TiO_2_	112.5	30	7.5
6	P-H-10T	70% UHMWPE + 20% HAP + 10% TiO_2_	105	30	15

The neat UHMWPE specimen (Sample P) was synthesized and evaluated under identical conditions to serve as a baseline control, enabling a direct quantitative assessment of the enhancements provided by the hybrid HAP and TiO _2_reinforcements against the standard unreinforced polymer matrix.

### Composite characterization

**Fig. 4:**
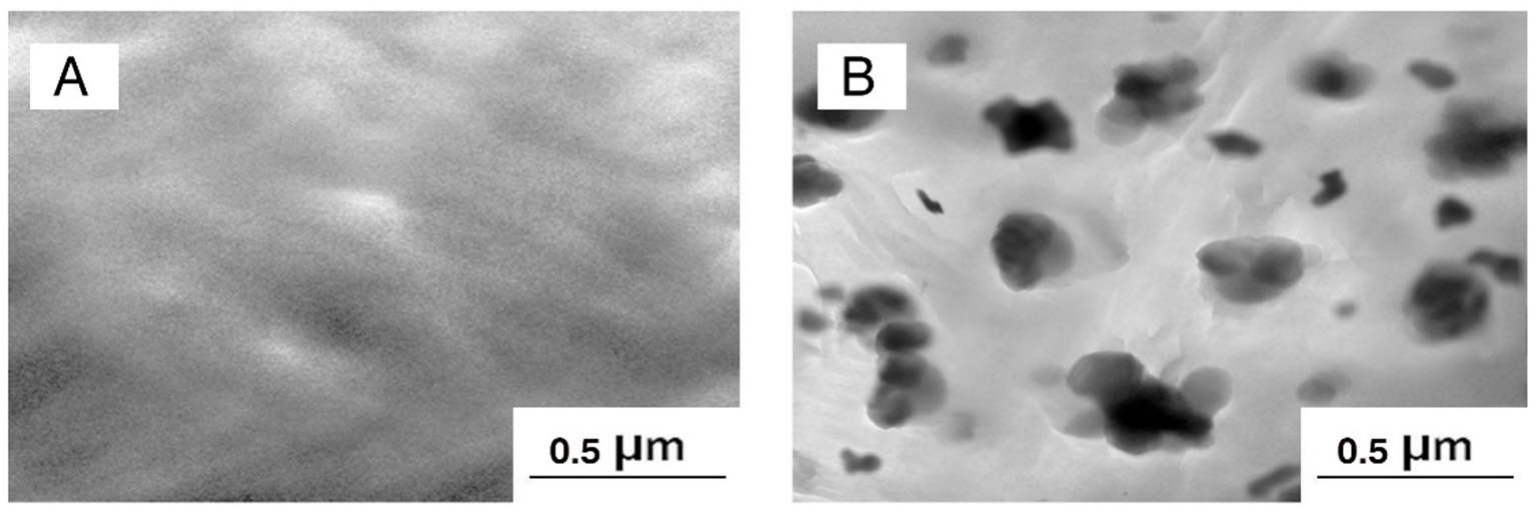
TEM images of (**A**) UHMWPE, and (**B**) UHMWPE + HAP+TiO_2_.

The transmission electron microscopy (TEM) technique can examine a specimen’s crystallographic structure, morphology, and even composition. The examination was carried out with a (JEM-2100 JEOL, Japan) in a range of 1000 to 2000 cm^− 1^ and a scanning resolution of 2 cm^− 1^. The TEM images show the dispersions of the HAP and TiO_2_ NPs in the UHMWPE matrix. Figure 4A shows the TEM image for UHMWPE as a blank, and Fig. 4B shows the TEM image for UHMWPE + HAP+TiO_2_ with 3 wt% TiO_2_, which has the best dispersion on the SEM images. The TEM images showed that the HAP and TiO_2_ NPs were well dispersed in the UHMWPE matrix and that the sample was free of aggregations.

**Fig. 5:**
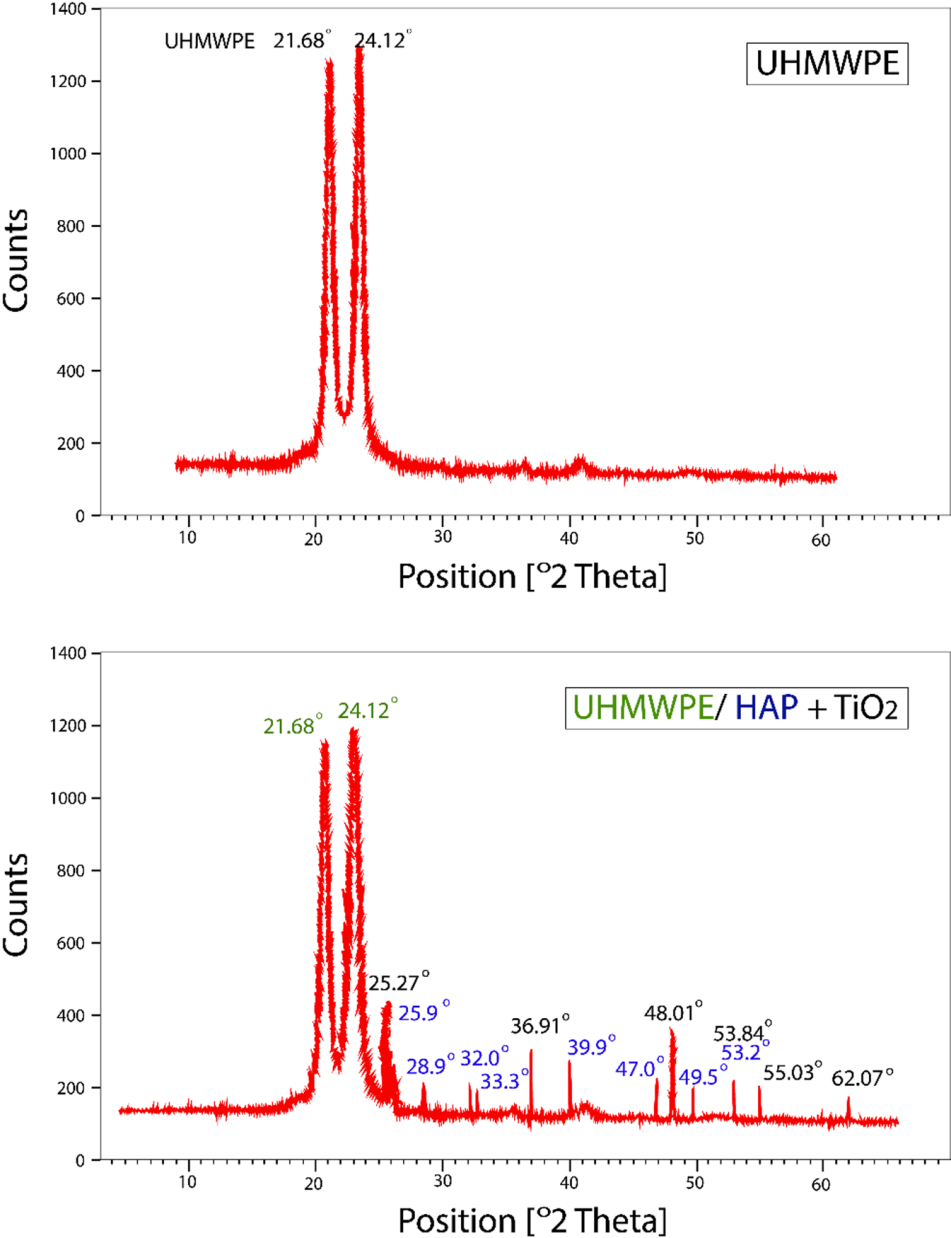
XRD analysis for neat UHMWPE and UHMWPE/HAP + TiO_2_.

An XRD analysis (D/MAX-2500 diffractometer, Rigaku, Japan) was conducted to examine the impact of TiO_2_ on the crystalline structure of UHMWPE. The crystalline atoms cause the incident X-ray beam to diffract in various directions. The phases present in the composite (UHMWPE/HAP + TiO_2_) were also defined. The diffraction patterns ranged from 10° to 70° on a 2θ scale. The characteristic peaks of UHMWPE were identified at 2θ values of 21.68^o^ and 24.12^o^, assigned to (1 1 0 and 2 0 0) planes^38^. The TiO_2_ nanoparticles exhibited peaks at 2θ values of 25.27^o^, 36.91^o^, 48.01^o^, 53.84^o^, 55.03^o^, and 62.07^o^, assigned to (1 0 1, 0 0 4, 2 0 2, 2 1 1, 2 2 0, 2 0 4) planes^39^. The anatase structure was confirmed by the robust diffraction peaks at 25.27° and 48.01°^40^. The HAP peaks were also well-defined in the XRD pattern at 25.9^o^, 28.9^o^, 32.2^o^, 33.3^o^, 39.9^o^, 47^o^,49.5^o^, and 53.2^o^, assigned to (0 0 2, 2 1 0, 2 1 1, 1 1 2, and 0 0 4) planes^41^. The XRD analysis for pure UHMWPE and UHMWPE/HAP + TiO_2_ is shown in Fig. 5.

### Wear tests

**Fig. 6:**
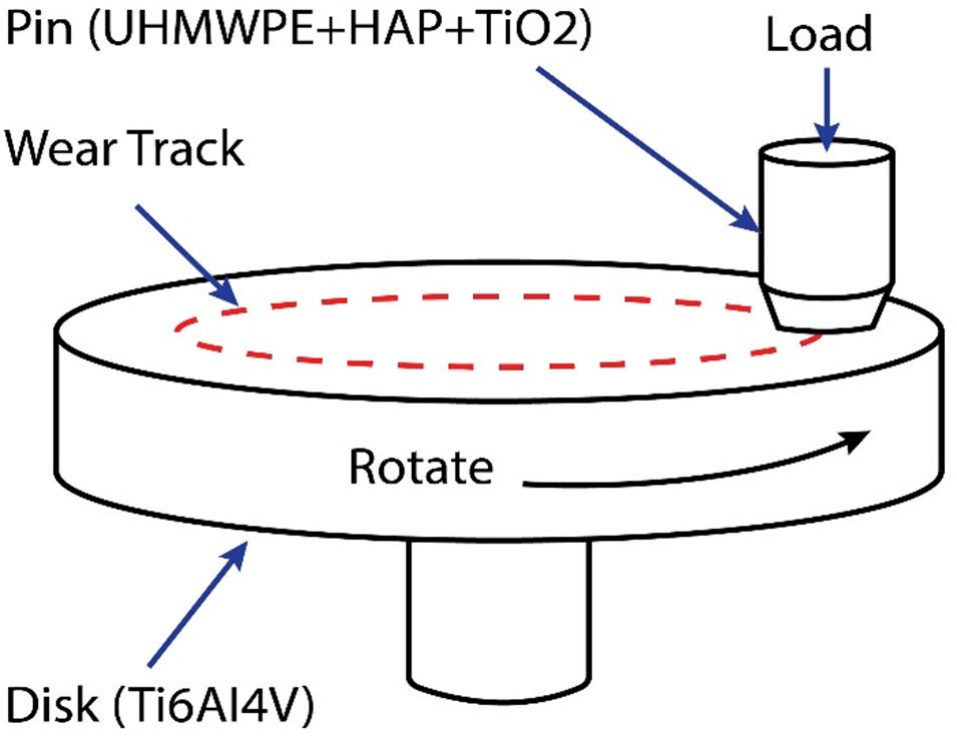
Pin and disk.

The wear tests were carried out using a pin-on-disk tribometer as shown in Fig. 6. The pin-on-disc configuration represents a simplified tribological model and does not fully replicate the complex kinematics of human joints^42^. The tribological test parameters were performed in accordance with ASTM F732, the standard test method for wear testing of polymeric materials used in total joint prostheses^43^. The counterface disc was fabricated from Ti6Al4V alloy (Grade 5), which typically exhibits a hardness in the range of approximately 32–36 HRC depending on processing conditions^44^, ensuring sufficient resistance to plastic deformation during sliding contact and maintaining stable tribological conditions throughout the wear tests. The average surface roughness (Ra) of the polished counterface disc was approximately 0.25 μm.

The pins had a cylindrical geometry with an initial diameter of 10 mm and were subsequently machined to a tapered contact end with a diameter of 3 mm in order to localize the contact region and maintain a controlled contact pressure during sliding. Prior to testing, the disc surface was cleaned with 100% ethanol and dried to eliminate the influence of previous experiments. Both the pins and disc were polished using silicon carbide abrasive paper (220 grit), which was selected to produce a controlled and repeatable surface condition suitable for comparative tribological evaluation without introducing excessive surface roughness variation.

After cleaning, the wear tests were conducted at 37 °C under both dry and lubricated conditions using simulated body fluid (SBF). The temperature was maintained using a thermostatically controlled closed-chamber wear testing device. In the experiments, a normal load of 20 N was applied, corresponding to a simplified contact pressure representative of physiological joint loading conditions associated with a subject body weight of approximately 75 kg^45^. Since the present study employed a simplified pin-on-disc configuration rather than a conformal joint geometry, Hertzian contact stress calculations were not considered directly applicable.

The effective wear-track radius of the disc was 60 mm, corresponding to a wear-track perimeter of approximately 377 mm. The experiments were conducted over a total sliding distance of 500 m at a linear sliding speed of 0.2 m/s, corresponding to a total test duration of approximately 42 min. Five samples from each composition were tested to improve statistical reliability, and the reported values represent the average results.

Wear performance was evaluated using wear volume loss as the primary wear parameter. The wear volume was measured using a micro-XAM three-dimensional surface profiler to minimize the influence of SBF absorption on the wear assessment of soaked samples. The following equation is then used to determine the wear rate:

1 $$W = \:\frac{V}{{F\times\:L}}\left( {{\mathrm{mm}}^{{\mathrm{3}}} {\mathrm{N}}^{{ - {\mathrm{1}}}} {\mathrm{m}}^{{ - {\mathrm{1}}}} } \right)$$

2 $${\text{V }} = {\text{ S }} \times {\text{ H }}\left( {{\mathrm{mm}}^{{\mathrm{3}}} } \right)$$

W stands for wear rate, V for wear volume (measured in mm^3^), F for applied normal load (measured in N), L for total sliding distance (measured in meters), S for cross-sectional area (measured in millimeters squared) of the samples’ wear zone, and H for overall length (wear distance). Finally, tribological performance and SEM pictures of the wear surfaces were used to study the wear performance of the UHMWPE/HAP + TiO_2_ composite.

### Fatigue test

Fatigue tests were conducted to evaluate the cyclic loading resistance of the UHMWPE/HAP/TiO_2_ composites under uniaxial tension–compression conditions using a servo-hydraulic fatigue testing machine (Instron 8801, USA). Cylindrical specimens with a diameter of 8 mm and a gauge length of 15 mm were prepared according to ASTM D7791-12 standard for polymer fatigue testing.

Tests were performed under stress-controlled mode with a sinusoidal waveform at two frequencies: 1 Hz and 3 Hz, representing low and moderate cyclic loading rates comparable to physiological joint motion. The stress ratio (R) was maintained at 0.1 (σ_min_/σ_max_ = 0.1) to simulate predominantly tensile cyclic conditions. Each specimen was tested at room temperature (25 ± 2 °C) until fracture or up to 10⁶ cycles, whichever occurred first.

The maximum applied stress (σ_max_) was varied to establish the S–N relationship between the applied cyclic stress and the number of cycles to failure (N_f_). Three specimens were tested for each composition and frequency, and the mean values were used to construct the S–N curves. The results showed that the composite containing 3 wt% TiO_2_ achieved the highest fatigue strength and lifetime, in agreement with wear performance trends.

### Antibacterial examination

The recommended method by the Clinical and Laboratory Standards Institute (CLSI, 2012) for evaluating the antibacterial activity of the samples in solid media was the Agar Well Diffusion Method. To activate the gram-positive *Staphylococcus aureus* and gram-negative *Escherichia coli* bacteria, BHI broth (Brain-Heart Infusion) was used, and the suspensions (BHI bacteria) were incubated at 37 °C in a bacteriological oven for 24 h. In Petri dishes, Mueller Hinton agar culture medium was arranged, and the bacterial suspensions were evenly distributed on the surface of the solid agar culture medium using the surface plating technique after solidification. The microorganisms were distributed evenly across the surface by sowing in all directions. In the solid agar culture medium, six circular holes with a diameter of 10 mm were made. In these holes, circular blend samples were deposited for microbiological analysis. After being incubated at 37 °C in a bacteriological oven for 24 h, the bacterial inhibition halo on the agar culture media was measured for the samples^17,46,47^. Furthermore, to quantitatively determine the bacterial reduction efficiency, the viable plate count method was employed. The composite specimens were immersed in the bacterial suspension (10^5^ CFU/mL) and incubated at 37 °C for 24 h. Following incubation, serial dilutions were performed, and the surviving bacteria were seeded onto nutrient agar plates. The colony-forming units (CFU) were counted after 24 h to calculate the precise antibacterial rate. The complete microbiological and counting protocols strictly aligned with standard clinical evaluation procedures reported in the literature^48^.

## Results and discussion

### Wear performance

**Fig. 7:**
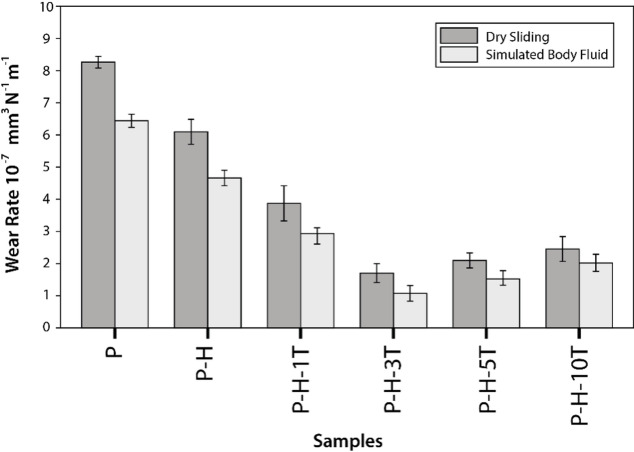
Wear rate of the samples under Dry and lubricating conditions.

**Fig. 8:**
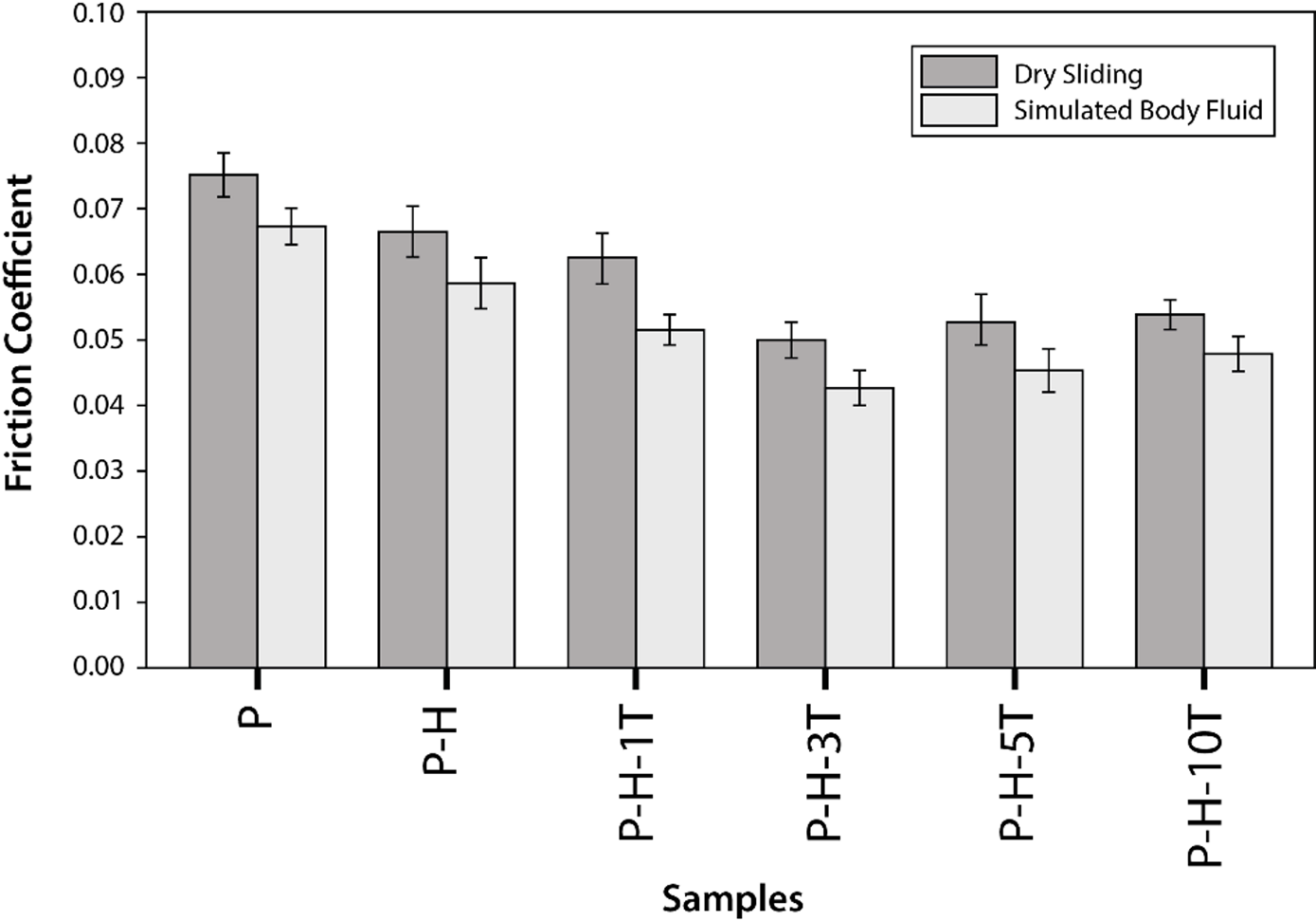
(COF) of the samples under Dry and lubricating conditions.

The wear tests were conducted on three different types of samples: pure/neat UHMWPE, UHMWPE combined with HAP, and UHMWPE with HAP and TiO₂, all of which were produced using the dispersing method. The dispersing method was selected to achieve improved nanoparticle distribution and clearer SEM characterization, enabling accurate determination of wear rate and coefficient of friction (COF). The results are presented in Fig. 7 (wear rate) and Fig. 8 (COF under dry and lubricated conditions).

As shown in Fig. 7, the P-H-3T composite exhibited the most stable tribological response under both dry and lubricated conditions. This behavior is associated with an optimal balance between nanoparticle dispersion and interfacial interaction within the UHMWPE matrix. At 3 wt% TiO₂, a more uniform distribution of HAP and TiO₂ nanoparticles is achieved, which enhances load transfer and promotes the formation of a compact and stable tribological layer. This layer reduces direct asperity interaction and limits third-body abrasion. In contrast, the increase in wear rate observed at higher TiO₂ contents (P-H-5T and P-H-10T) may be attributed to nanoparticle agglomeration and non-uniform distribution within the polymer matrix. Such agglomeration can lead to localized stress concentrations and reduced interfacial efficiency, which adversely affect wear resistance. However, further detailed microstructural characterization would be required to fully confirm this interpretation. Therefore, the saturation level of the UHMWPE matrix with TiO₂ nanoparticles can be considered to occur at approximately 3 wt%, where minimum wear rate and COF are obtained. The observed reduction in both wear rate and COF suggests that the tribological improvement is governed by a common interfacial stabilization mechanism. The formation of a compact tribological transfer layer at the sliding interface reduces direct asperity interaction, thereby simultaneously decreasing frictional resistance and material removal. Consequently, the reduction in COF contributes to limiting surface damage accumulation and abrasive wear under both dry and lubricated conditions. Table 3. Shows the summary of wear rate and coefficient of friction (COF) values for UHMWPE/HAP/TiO₂ composites under dry and lubricated (SBF) conditions.

**Table 3:** Summary of wear rate and coefficient of friction (COF) values for UHMWPE/HAP/TiO₂ composites under dry and lubricated (SBF) conditions.

Sample	Wear Rate (Dry) ×10⁻⁷ (mm³ *N*⁻¹ m⁻¹)	Wear Rate (SBF) ×10⁻⁷ (mm³ *N*⁻¹ m⁻¹)	COF (Dry)	COF (SBF)
P	8.2	6.5	0.078	0.070
P-H	6.1	5.2	0.068	0.060
P-H-1T	4.2	3.1	0.062	0.052
P-H-3T	1.75	1.07	0.050	0.042
P-H-5T	2.3	1.8	0.056	0.047
P-H-10T	2.7	2.1	0.058	0.049

**Fig. 9:**
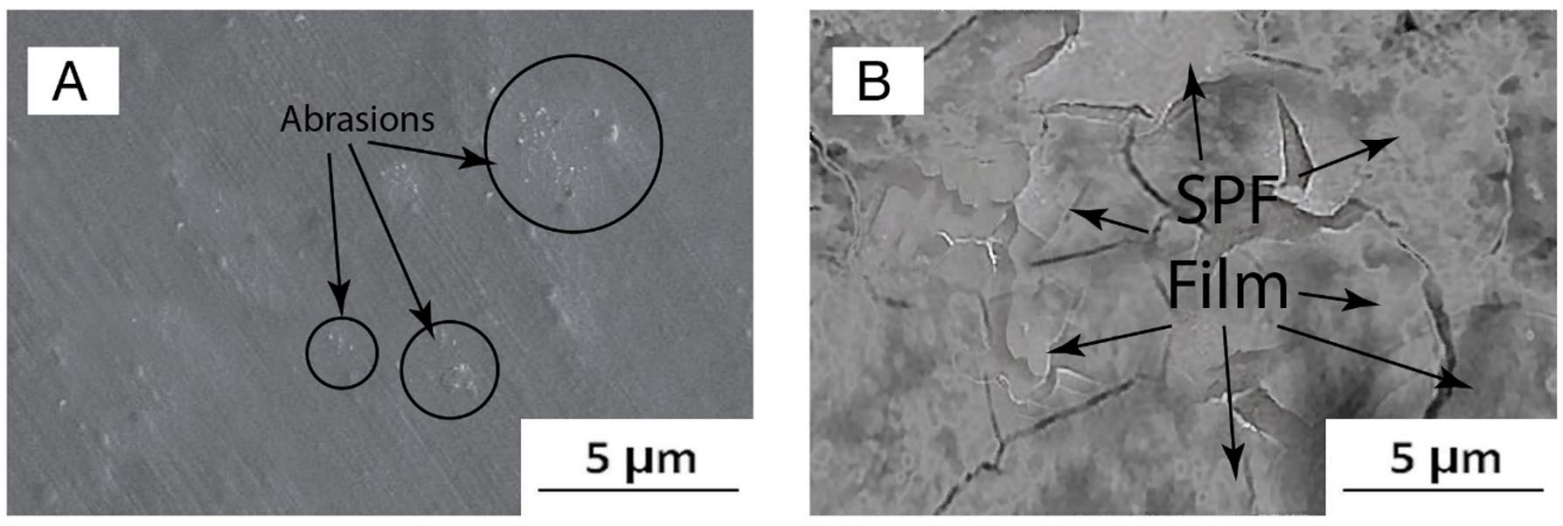
SEM images of the wear surfaces of sample P-H-3T under (**A**) Dry sliding, (**B**) SBF lubricant.

After conducting wear tests, SEM analysis was used to examine the surface morphology of the composite pins. The dominant wear mechanism observed was abrasive wear, characterized by scratches, grooves, and micro-cracks formed under point contact loading. Frictional heating during sliding contributes to localized thermal softening along the wear track, facilitating material removal and surface damage^3^. Figure 9 presents SEM images of the P-H-3T sample under dry and lubricated conditions. Under dry sliding (Fig. 9a), the surface exhibits limited abrasion features, indicating improved resistance to severe surface damage. Under lubricated conditions (Fig. 9b), the formation of a continuous SPF-derived film is observed, which adheres to the surface and forms a dense protective layer. This tribo-film reduces direct surface contact and enhances wear resistance. The presence of micro-cracks within the film, attributed to residual tensile stresses, is also observed^49^, but does not significantly compromise the protective behavior of the layer.

SEM characterization was primarily focused on the P-H-3T composite because it exhibited the optimum tribological performance among all investigated compositions under both dry and lubricated conditions. The selected micrographs were intended to identify the dominant wear mechanisms associated with the best-performing composite and to correlate surface morphology with the observed wear and friction behavior. Nevertheless, comparative wear trends among the remaining samples were evaluated quantitatively through wear rate and COF measurements presented in Figs. 7 and 8. In addition, five samples from each composition were tested under identical conditions, and the reported results represent average values to ensure experimental repeatability and reliability.

In addition to the quantitative wear results, the role of wear debris and lubrication regime must be considered to better interpret the tribological response and its biomedical relevance. In dry sliding conditions, the generated wear debris may accumulate at the contact interface, acting either as third-body abrasives that accelerate surface damage or as compacted transfer layers that stabilize friction, depending on particle morphology and distribution^15^. The improved performance of the P-H-3T composite is likely associated with the generation of finer and more stable debris, which reduces severe third-body abrasion and limits fatigue-induced microcrack initiation.

Under simulated body fluid (SBF/SPF) lubrication, the tribological mechanism shifts toward boundary or mixed lubrication regimes, where a fluid film partially separates the contact surfaces. This reduces direct asperity interaction and facilitates debris removal from the contact zone, thereby minimizing abrasive and fatigue wear^16^. Furthermore, the formation of a stable tribo-film observed in SEM micrographs suggests enhanced surface protection, consistent with findings reported for polymer-based biomedical composites under physiological lubrication conditions^50^.

From a biomedical perspective, evaluating both dry and lubricated regimes provides a broader understanding of material performance. While dry testing represents severe contact conditions, SBF lubrication better approximates physiological environments. Therefore, the selected test conditions provide useful insight into fatigue–wear interactions under simplified in vitro conditions, although they do not fully replicate the complex kinematics and loading conditions of in vivo joint systems.

When comparing the findings of this study with previous UHMWPE-based systems incorporating different reinforcements such as GNP^11^, CNTs^51^, SiC^9^, PTFE^3^, GO^8^, EGCG^3^, and Ag/ZnO^15^, the present hybrid composite demonstrates a distinct performance advantage. As summarized in Table 4, the UHMWPE/20 wt% HAP/3 wt% TiO₂ composite exhibits a low COF (0.05) under dry sliding conditions. However, such comparisons should be interpreted with caution due to differences in test conditions, counterface materials, and operating environments.

The improved performance of the present system can be attributed to a synergistic reinforcement mechanism. HAP contributes to enhanced load distribution and interfacial stiffness, reducing micro-fatigue crack initiation, while TiO₂ promotes surface stabilization and controlled third-body behavior, facilitating the formation of a stable tribological layer. Unlike single-filler systems, this hybrid structure enables simultaneous reduction of friction and enhancement of mechanical integrity.

Moreover, the stabilization of the sliding interface plays a critical role in maintaining surface integrity. Reduced debris accumulation and limited surface roughening decrease localized stress concentrations, which are primary sites for fatigue crack initiation. Therefore, the tribological improvements observed in the P-H-3T composite directly contribute to enhanced fatigue resistance in biomedical applications.

**Table 4:** Literature COF comparison.

Material	Coefficient of Friction	References
	Dry Sliding	
UHMWPE + HAP+TiO_2_	0.05	This work
UHMWPE + GNP+HAP	0.075	^11^
UHMWPE+ CNTs	0.1	^50^
UHMWPE + SiC	0.3	^9^
UHMWPE+ PTFE	0.063	^14^
UHMWPE + GO	0.095	^8^
UHMWPE+ EGCG	0.15	^15^
UHMWPE + Ag+ZnO	0.1	^13^

### Fatigue performance

**Fig. 10:**
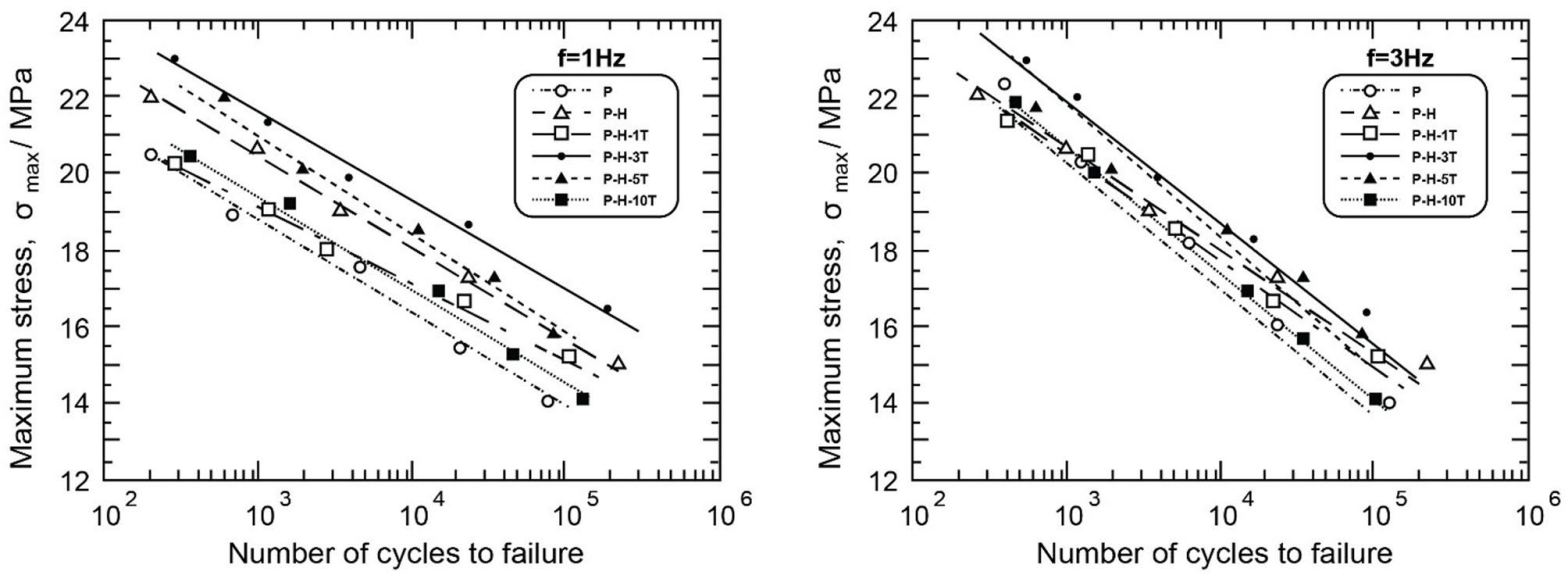
S–N curves showing the fatigue behavior of UHMWPE/HAP/TiO2 composites at loading frequencies of 1 Hz and 3 Hz. The P-H-3T sample (3 wt% TiO_2_) demonstrated the highest fatigue strength and endurance life.

Fatigue tests were performed at loading frequencies of 1 Hz and 3 Hz to investigate the cyclic behavior of the UHMWPE/HAP/TiO₂ composites. The S–N curves (Fig. 10) exhibit a typical logarithmic relationship between applied stress (σ_max) and number of cycles to failure (Nf), characteristic of viscoelastic polymeric materials.

The P-H-3T composite exhibited the highest fatigue strength at both frequencies, sustaining higher stress levels for equivalent fatigue lives compared to other samples. This improvement is attributed to the uniform dispersion of TiO₂ and HAP nanoparticles, which enhances interfacial bonding and promotes efficient stress transfer. As a result, stress concentrations are reduced, delaying crack initiation and slowing crack propagation under cyclic loading. At higher TiO₂ contents (5 wt% and 10 wt%), particle clustering becomes more pronounced, leading to localized stress amplification and early crack initiation. Consequently, fatigue performance deteriorates. Additionally, specimens tested at 3 Hz showed slightly higher fatigue limits than those at 1 Hz, indicating a strain-rate-dependent strengthening effect associated with cyclic hardening.

The wear results demonstrated that the P-H-3T composite exhibits the lowest wear rate under both dry and lubricated conditions, while fatigue testing confirmed its superior cyclic durability. This concurrent improvement can be explained through a surface-controlled damage mechanism. In UHMWPE-based materials, fatigue failure is often initiated at surface or near-surface defects generated during sliding contact. For the optimally reinforced P-H-3T composite, the formation of a stable tribological layer reduces surface degradation, including grooves, debris accumulation, and micro-defects. This preservation of surface integrity limits the formation of crack initiation sites under cyclic loading. Consequently, the onset of fatigue damage is delayed, and crack growth is suppressed.

Furthermore, the homogeneous distribution of TiO₂ and HAP enhances internal stress redistribution and promotes crack deflection, increasing the energy required for crack propagation. Therefore, the improved fatigue performance is not solely a result of reduced wear magnitude, but rather a consequence of combined surface stabilization and internal stress homogenization mechanisms within the hybrid composite. The applied stress ratio and cyclic frequency were selected to simulate simplified physiological loading conditions. However, it should be noted that actual in vivo joint loading involves complex multiaxial stresses and variable loading conditions that cannot be fully replicated in the present experimental setup.

### Antibacterial properties

**Fig. 11:**
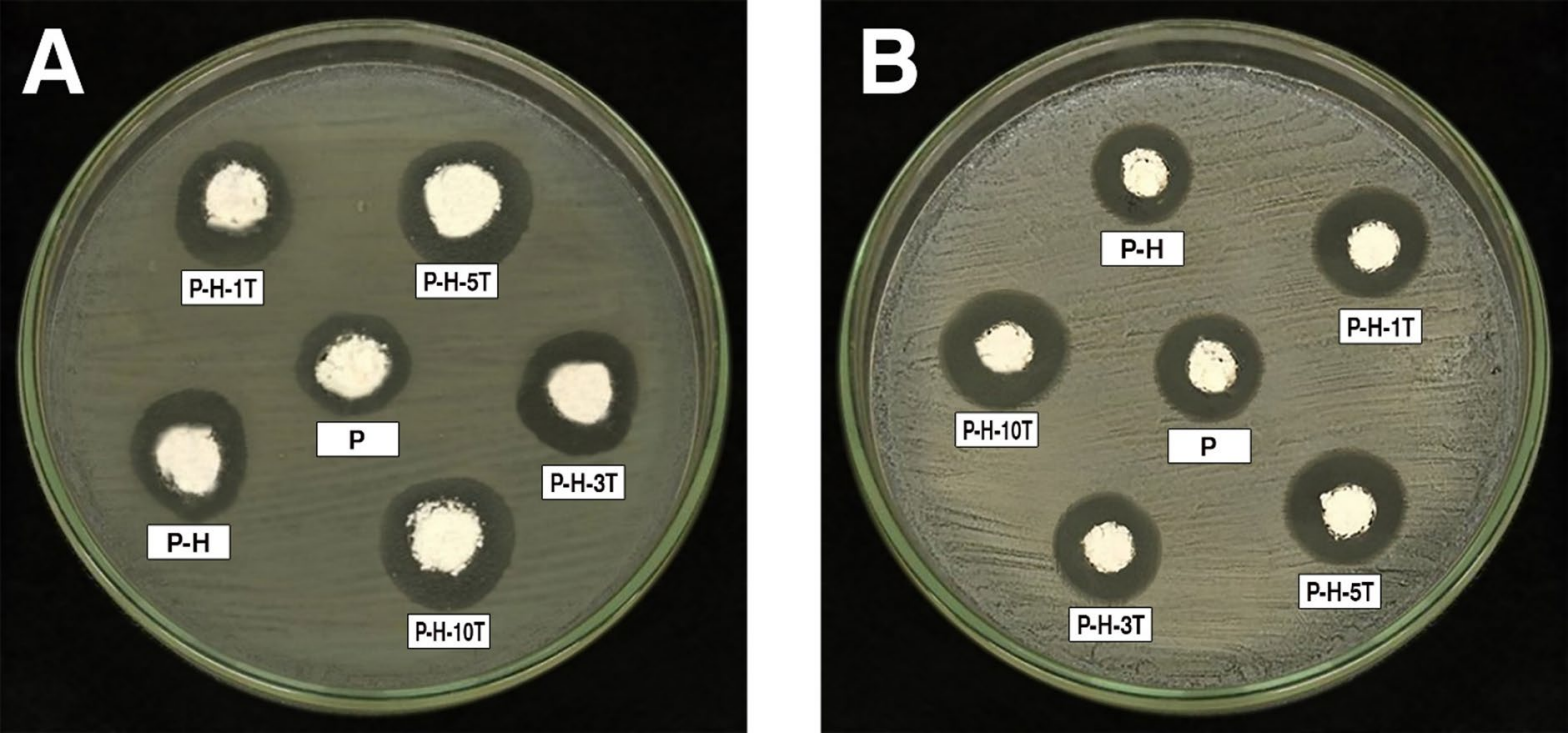
Bacterial inhibition area, (**A**) *Escherichia coli*, (**B**) *Staphylococcus aureus*.

Figure 11 illustrates the antibacterial assessment performed using the agar disc diffusion method against *Staphylococcus aureus* and *Escherichia coli*, with the corresponding zone diameters and relative antibacterial rates summarized in Table 5. Sample P was utilized as the control to evaluate the enhanced efficiency of the modified composites. As observed, all samples exhibited distinct and measurable inhibition zones. The neat sample (P) showed inhibition zones of 20 mm and 24 mm against *E. coli* and *S. aureus*, respectively. Notably, the incorporation of TiO₂ nanoparticles led to a progressive and significant increase in the inhibition zone diameters, reaching maximum values of 29 mm (*E. coli*) and 31 mm (S. aureus) for the P-H-10T sample. This corresponds to a relative antibacterial enhancement rate of 45.0% and 29.2%, respectively, compared to the control sample.

However, an increase in the measurable inhibition diameter was observed in samples containing TiO₂ nanoparticles, indicating enhanced antibacterial effectiveness compared to neat UHMWPE. The improved antibacterial performance is more pronounced against *Staphylococcus aureus* (Gram-positive) than *Escherichia coli* (Gram-negative), which can be attributed to structural differences in their cell walls. Gram-negative bacteria possess an additional outer membrane that provides higher resistance to oxidative and nanoparticle-induced stress.

The antibacterial activity of TiO₂ is mainly associated with its ability to generate reactive oxygen species (ROS), such as hydroxyl radicals and superoxide ions, particularly under light exposure. These ROS can induce oxidative damage to bacterial cell membranes, disrupt protein structures, and cause DNA fragmentation, ultimately leading to bacterial inactivation. Additionally, direct nanoparticle–cell membrane interaction may contribute to membrane destabilization. Thus, while solid diffusion limitations reduce halo visibility, the incorporation of TiO₂ enhances the intrinsic antibacterial functionality of the composite material.

Moreover, the reduction in wear debris generation may indirectly support antibacterial performance by limiting surface irregularities that facilitate bacterial adhesion and biofilm formation. Thus, the hybrid reinforcement strategy provides a multifunctional synergy between mechanical durability and biological resistance. These findings are consistent with recent reports on Ti-based biomaterials, which emphasize ROS-mediated antibacterial mechanisms and membrane-disruptive effects as key contributors to bacterial inactivation^52^. Furthermore, oxidative surface interactions induced by TiO₂ may influence host–bacteria responses and reduce biofilm persistence under physiological conditions, as discussed in recent immunological investigations^53^.

**Table 5:** Bacterial inhibition zone diameters.

Samples	Inhibition zone diameter (mm)	Antibacterial Rate (%)	Inhibition zone diameter (mm)	Antibacterial Rate (%)
	*Escherichia coli*		*Staphylococcus aureus*	
P (Control)	20	–	24	–
P-H	23	15.0%	24	0.0%
P-H-1T	24	20.0%	26	8.3%
P-H-3T	25	25.0%	27	12.5%
P-H-5T	27	35.0%	29	20.8%
P-H-10T	29	45.0%	31	29.2%

Antibacterial Rate (%) represents the percentage increase in the inhibition zone diameter relative to the control sample (P).

## Conclusions

This study demonstrates that incorporating TiO₂ nanoparticles into the UHMWPE/HAP system significantly enhances tribological, fatigue, and antibacterial performance. The optimal composition was achieved at 3 wt% TiO₂, which provided the best balance between wear resistance, reduced coefficient of friction, and improved fatigue durability under both dry and simulated physiological conditions.

The enhanced performance is attributed to synergistic reinforcement mechanisms, including improved load transfer, crack propagation resistance, stable tribo-film formation under lubrication, and TiO₂-driven antibacterial activity. However, higher TiO₂ contents led to particle agglomeration, negatively affecting mechanical stability.

Overall, the UHMWPE/HAP/3 wt% TiO₂ composite offers multifunctional improvement combining mechanical durability and biological resistance, highlighting its strong potential for long-term orthopedic bearing and joint replacement applications.

This study systematically investigated the influence of hybrid HAP and TiO_2_ nanoparticles on the physical, mechanical, tribological, and biological performance of UHMWPE composites for load-bearing orthopedic implants. The principal conclusions derived from this work are summarized as follows:

1. The thermo-mechanical fabrication process successfully yielded high-density hybrid composites, with a controlled and significant improvement in surface microhardness up to the P-H-10T formulation.
2. Tribological evaluations revealed a direct proportionality between the coefficient of friction (COF) and the volumetric wear rate under both dry and fluid environments; the incorporation of an optimal 3 wt% TiO_2_ minimized deep cutting and plowing, resulting in the highest wear resistance.
3. Statistical fatigue life modeling using the two-parameter Weibull distribution demonstrated that hybrid reinforcements substantially extended the B_50_ median life, with the 3 wt% TiO_2_ formulation achieving the highest reliability and lowest data scattering $\:(\beta\:=\:2.45)$.
4. Microbially, the hybrid composites exhibited robust, concentration-dependent antibacterial efficacy against both Gram-positive (*S. aureus*) and Gram-negative (*E. coli*) bacteria, reaching optimal reduction rates at higher nanoparticle loadings.
5. Structurally and functionally, the hybrid composite reinforced with 3 wt% TiO_2_ presents the most optimized and synergistic balance of wear mitigation, dynamic fatigue endurance, and antimicrobial protection, rendering it a highly promising candidate for synthetic joint prostheses.

## Data Availability

All of the material is owned by the authors and no permissions are required.The datasets used and analysed during the current study available from the corresponding author on reasonable request.
